# Supplementary material for: Exogenous Iron Increases Fasciocidal Activity and Hepatocellular Toxicity of the Synthetic Endoperoxides OZ78 and MT04
Source: Int J Mol Sci. 2019 Oct 1;20(19):4880. doi: 10.3390/ijms20194880 (PMC6801819; doi:10.3390/ijms20194880)
Supplement: Supplementary file 1 [file ijms-20-04880-s001.pdf]

Supplementary Table 1: Effect of AS, OZ78 and MT04 on mouse liver histology. Mice were treated by oral gavage with single doses of 200, 400 and 800 mg/kg of AS, OZ78 or MT04. Livers were obtained 8 hours after oral administration.

Treatment	Dose	Mouse	Histopathologic observation
**Control**		1	No lesions
		2	No lesions
		3	No lesions
		4	No lesions
		5	Focal hemorrhage, severe acute congestion, syncytial cells
		6	No lesion
		7	Steatosis and syncytial cells
**AS**	200 mg/kg	1	No lesions
		2	No lesions
		3	No lesions
	400 mg/kg	1	No lesions
		2	No lesions
		3	Beginning nodular regeneration
	800 mg/kg	1	Steatosis
		2	Small area of confluent necrosis
		3	No lesions
**OZ78**	200 mg/kg	1	No lesions
		2	Slight hydropic degeneration
		3	Slight hydropic degeneration
		4	No lesions
	400 mg/kg	1	Slight hydropic degeneration
		2	Passive congestion
		3	Nuclear polymorphism
		4	Slight hydropic degeneration
	800 mg/kg	1	Slight hydropic degeneration
		2	No lesions
		3	Passive congestion
		4	Slight hydropic degeneration
**MT04**	200 mg/kg	1	No lesions
		2	No lesions
		3	No lesions
		4	Nuclear polymorphism
	400 mg/kg	1	Nuclear polymorphism
		2	Nuclear polymorphism
		3	No lesions
		4	No lesions
	800 mg/kg	1	No lesions
		2	Slight hydropic degeneration
		3	Slight hydropic degeneration and nuclear polymorphism
		4	Slight hydropic degeneration and nuclear polymorphism
